# Complete genome sequence of a sapovirus from a child in Zhejiang, China

**DOI:** 10.1007/s11262-016-1343-9

**Published:** 2016-04-28

**Authors:** Xiaohong Zhou, Yi Sun, Xiaochun Shang, Jian Gao, Xueqin Zhao, Huiqun Shuai, Rui Zhang, Yanjun Zhang

**Affiliations:** 1 Xiacheng District Center for Disease Control and Prevention, Hangzhou, Zhejiang China; 2 Zhejiang Provincial Center for Disease Control and Prevention, 3399 Binsheng Road, Hangzhou, 310051 Zhejiang China

**Keywords:** Sapovirus, Genome, Phylogeny

## Abstract

Although *Sapovirus* (*Caliciviridae*) has been accepted as one of the causes of acute gastroenteritis worldwide, little is known about the genetic characteristics of the whole genome of sapoviruses in China, especially those that infect humans. Here we report the complete genome sequence of a sapovirus strain, Human/Zhejiang1/2015/China, obtained from a child with acute gastroenteritis in Hangzhou, Zhejiang Province, China. Samples were collected and delivered to the CDC laboratories and were detected by RT-PCR. Sanger sequencing was used to obtain the full genome and molecular characterization of the genome was determined. A phylogenetic analysis of the genome was also performed. The results indicated that Human/Zhejiang1/2015/China belongs to Genogroup I. No recombination events were detected. This is the first complete sequence from a child to be reported in China. The sequence information is important for surveillance of this emerging gastrointestinal infection.

**Electronic supplementary material:**

The online version of this article (doi:10.1007/s11262-016-1343-9) contains supplementary material, which is available to authorized users.

## Introduction

*Sapovirus* (SAV; in the family *Caliciviridae*), causes acute gastroenteritis in both humans and animals. Major clinical symptoms include diarrhea and vomiting with constitutional symptoms such as nausea, chills, and myalgia. Compared to Norovirus, SAV infections are less common and are known to cause disease primarily in children, usually those under the age of 5 years [1]. SAV has received wide attention in public health studies because SAV has recently been a causative agent of gastroenteritis in people of all ages in both confined outbreaks and in sporadic cases [1, 2]. It has also been reported in outbreaks in long-term care facilities for the elderly [3]. Mortality is rare and the symptoms are generally mild. No licensed vaccines or antivirals are available for SAV infections.

Many outbreaks caused by foodborne transmission of SAV have been reported since 1976 in England, where SAV particles were first detected in human diarrheic stool samples [4]. The largest foodborne SAV outbreak recorded, which involved 665 people, was in Japan in 2010 [5]. Many worldwide studies have reported that SAV can be divided into several genogroups, ranging from GI to GV [6–8]. Recent studies found that SAV genogroup VI in isolated fecal samples from diarrheic pigs [9]. Different genogroups result in the infection of different hosts; GI, GII, GIV, and GV infect humans and other primates, whereas GIII infects swine. Furthermore, there are at least eight genotypes of GI, five of GII, and one each of GIII, GIV, and GV. Most SAV detected in humans belong to GI and GII [10, 11].

Although SAV has been accepted as one of the causes of acute gastroenteritis worldwide, little is known about the genetic characteristics of sapoviruses in China based on whole genome analysis, especially about genotypes infecting humans, due to infrequent detection and difficulties in culturing and sequencing, especially whole genome sequencing. Here we report the complete genome sequence of SAV strain Human/Zhejiang1/2015/China obtained in late 2014 from a child with acute gastroenteritis in Hangzhou, Zhejiang Province, China. Phylogenetic analysis was performed for comparison with other genogroups/genotypes reported previously. The purpose of this study was to characterize and present the first full-length genome of SAV as fundamental information in mainland China.

## Materials and methods

Samples were collected from a patient who had acute gastroenteritis in a kindergarten in the Xiacheng district of Hangzhou on December 25 and 26, 2014. Samples were delivered to the CDC laboratories and were made into 10 % (w/v) in phosphate-buffered saline suspensions (PBS) and centrifuged at 2000×*g* for 10 min. Viral RNA was extracted from 200 µl of the supernatant using a Qiagen RNeasy Mini kit (Qiagen) according to the manufacturer’s instructions. Eluted RNA was stored at −80 °C until use. RNA was detected by reverse transcription PCR with SAV universal primers (SV-F13, SV-F14, SV-R13, and SV-R14) using the following thermal cycling profile: 42 °C for 15 min; 95 °C for 3 min; 35 cycles of 95 °C for 30 s, 48 °C for 30 s, and 74 °C for 45 s according to the previous study of Okada et al. [14]. Full genome Sanger sequencing was performed according to the study by Liu et al. [15].

Geneious 4.8.3 (www.geneious.com) was used to assemble and check the sequences of this SAV, Human/Zhejiang1/2015/China. For the VP1 region and the whole genome, we performed multiple alignments with data matrixes of other sequences downloaded from GenBank for each segment, respectively. We blasted the homology sequences in NCBI for both the VP1 region and the genome, then checked for variant positions in amino acid sequences using MEGA 6.0 (www.mega.software.informer.com) for the whole genomic range. Dataset-specific models that were selected using the Akaike Information Criterion in Modeltest 3.7 were analyzed for each matrix [16]. Maximum likelihood (ML) analysis was processed in RAxML v7.2.8 using the VP1 region and genome, respectively (http://sco.h-its.org/exelixis/software.html). Optimal ML trees and bootstrap percentages (BP) were estimated in the same run. The ML BP values were obtained from 1000 BP replicates using the rapid BP algorithm. Both Simplot v3.5.1 and RDP 4.26 analysis with default set were used for detecting recombination event in this strain [17, 18].

## Results and discussion

This has been the first genome obtained from a child in China, in contrast to 47 complete human/porcine sapovirus sequences available in GenBank till the data published. The full genome sequence of the SAV from the patient was deposited in GenBank with accession number KT327081. The SAV obtained was a positive-sense, single-stranded RNA 7441 bp in size with a 3′-end poly (A) tail. Its 5′-UTR was 9 bp long; 3′-UTR was 94 bp long. The genome contained three open reading frames (ORFs). ORF1 encodes the nonstructural proteins (NS) from 10 to 6853 bp. ORF2 is a minor structural protein VP2 (from 6848 to 7347 bp). ORF3 is a hypothetical protein CDS from 5176 to 5662 bp, which has not been clearly defined [12]. The major capsid protein VP1, from 5164 to 6853 bp, was embraced in ORF1, which followed the seven nonstructural proteins from NS1 to NS7 in the 5′ to 3′ direction (supplementary materials). The NS pattern of ORF1 in our sequence was similar to the GI.1 Manchester strain (GenBank accession: X86560) except for one putative cleavage site between NS5 (VPg) and NS6–NS7 (Protease-RdRp), which was E/A rather than E/G, as mentioned in Oka’s review [13]. Typical amino acid motifs were also detected in our sequence, for example: GAPGIGKT from 480 to 487 amino acid (aa) in NS3, KGKTK, and DDEYDE from 941 to 945 aa and 962–967 aa in NS5, respectively. There were three PPG motifs in the VP1 region, which differed from the reference stain of GI.1 Manchester. The conserved GWS motif in the VP1 region was also detected in our sequence, which proved strictly characteristic of caliciviruses [13]. Chanthaburi-74/2004/Thailand (GenBank accession: AY646854) showed the highest query cover and percent nucleotide identity with our sequence based on the whole genome. We therefore compared amino acid substitutions in the coding regions (mainly including ORF1 and ORF2) in the two sequences (Table 1). Twenty-two variants were found in the nonstructural region while one and two substitutions were discovered in the VP1 and VP2 areas, respectively.

**Table 1:** Unique amino acid changes observed in Human/Zhejiang1/2015/China compared to Chanthaburi-74/2004/Thailand (AY646854)

	Nonstructural proteins																						VP1	VP2	
Polypeptide position	2	20	45	49	63	111	134	141	181	658	680	681	692	1082	1094	1179	1342	1388	1402	1484	1537	1590	1729	139	146
Chanthaburi-74/2004/Thailand	A	I	F	M	E	R	T	N	V	A	R	P	A	I	V	S	Q	K	V	V	N	D	P	P	S
Human/Zhejiang1/2015/China	V	V	Y	V	K	K	A	P	G	T	K	S	T	V	I	N	H	T	I	I	S	E	S	S	L

**Fig. 1:**
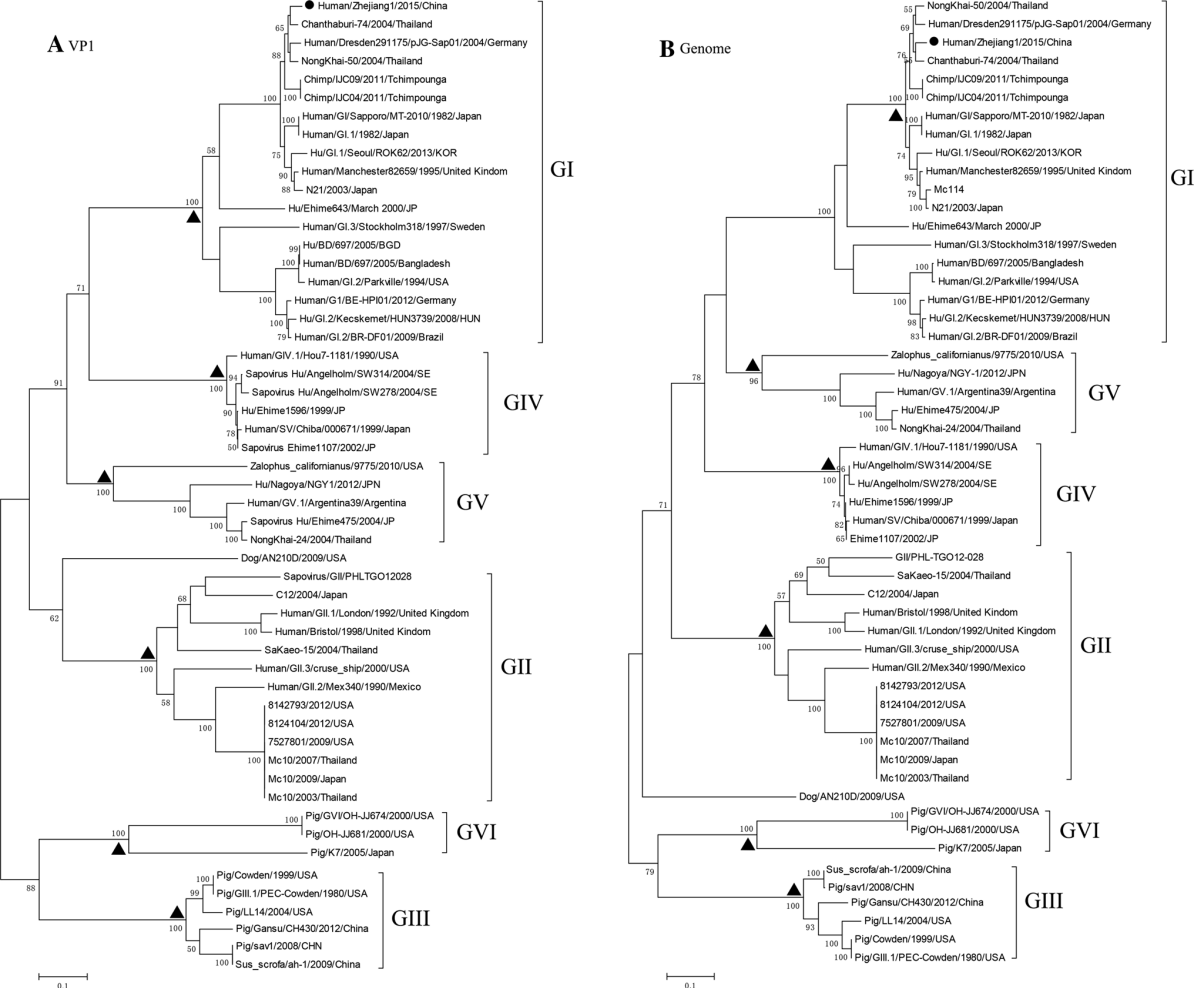
Maximum likelihood trees for sapovirus based on VP1 region and whole genome, respectively. BP values based on 1000 replicates are indicated above *branches*. *Solid circles* in each tree indicate sequences obtained in our study. *Triangles* at nodes indicate monophyletic clades of different genogroups. **a** VP1 tree, **b** genome tree

To better classify the sapovirus, we searched the NCBI database and found Hu/GI.1/8743/Maizuru/2008/JPN (HM030922) to be a close match in the VP1 region, while Chanthaburi-74/2004/Thailand (AY646854) was close to the whole genome. In these two maximum likelihood trees with Bootstrap percentages assigned, different genogroups, such as GI, GII, GIII, GIV, GV, and GVI, were reciprocally monophyletic with clades receiving 100 % BP support, with the exception of the GV clade in the genomic ML tree which had a BP value of 96 % (Fig. 1a, b). Our sequence, Human/Zhejiang1/2015/China, was in the GI clade and shared a relative high homology with Chanthaburi-74/2004/Thailand in both trees. The results of Blast from NCBI indicated sapovirus Human/Zhejiang1/2015/China to be in genogroup I/genotype 1 (G1.1). The phylogenetic structure in both ML trees was basically similar, although there was an inconsistency in that either GIV or GV could be the closest sister group of GI according to the VP1 region and the whole genome (Fig. 1a, b). Several possible scenarios may have led to this phenomenon, such as incomplete lineage sorting between genes and genome. We are further analyzing this hypothesis. This study, focused on the interior of the GI clade, indicated that Human/Zhejiang1/2015/China is not a recombinant, since both trees showed the strain we obtained to be clustered in a similar GI clade. This finding was subsequently substantiated by RDP analysis and Simplot (supplementary materials). Further studies are underway with additional viruses in the family *Caliciviridae*, including norovirus, based on surveillance programs of foodborne infections established by the national health and family planning commission of China.

## Conclusions

This first genome sequence of SAV from a child in Zhejiang, China, provides genetic characteristics of SAV infections in humans in the eastern China region. Based on complete capsid gene sequences from the phylogenetic results we determined that this SAV, Human/Zhejiang1/2015/China, belongs to GI, which is consistent with previous reports that GI is known to infect humans. Recombination was not detected. Our SAV sequence may help in both surveillance and in understanding the characterization of emerging gastroenteritis infections in China.

## Electronic supplementary material

Below is the link to the electronic supplementary material.

Supplementary material 1 (TIFF 711 kb)

Supplementary material 2 (TIFF 2352 kb)

Supplementary material 3 (DOC 82 kb)
