# Supplementary figures and images for: Complete genome sequence of a sapovirus from a child in Zhejiang, China
Source: Virus Genes. 2016 Apr 28;52(5):706–10. doi: 10.1007/s11262-016-1343-9 (PMC5002278; doi:10.1007/s11262-016-1343-9)

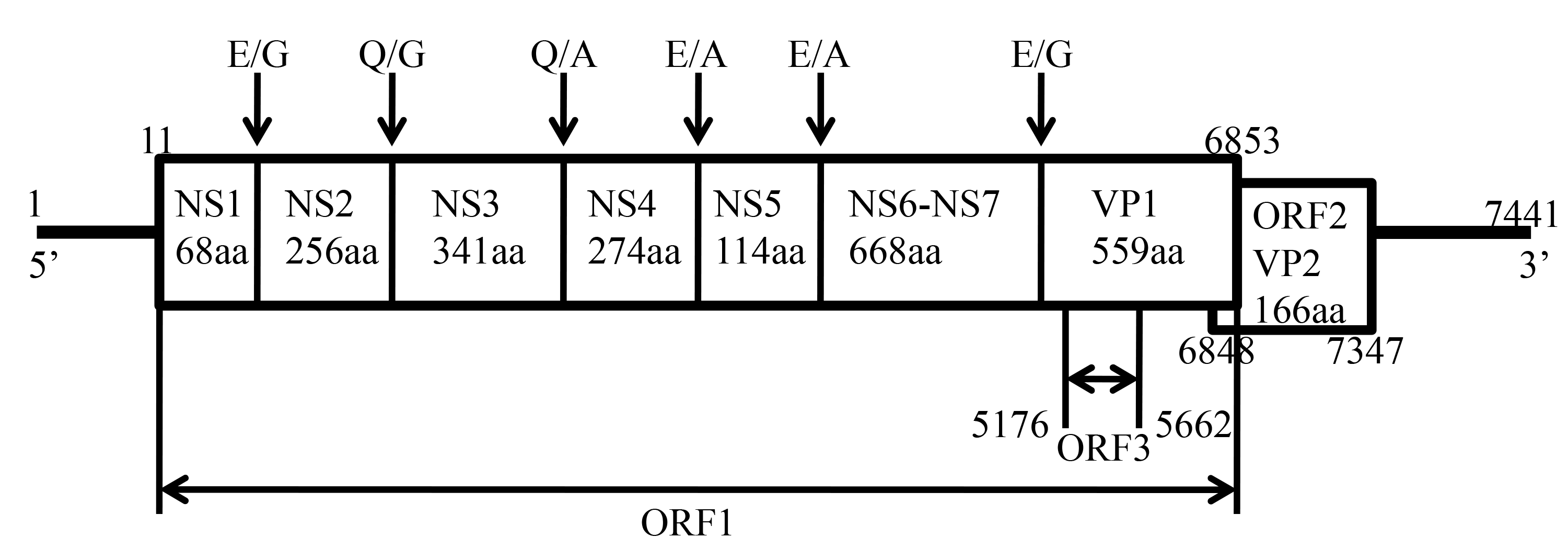

Supplement: Supplementary file 1 — Supplementary material 1 (TIFF 711 kb) [file 11262_2016_1343_MOESM1_ESM.tif]

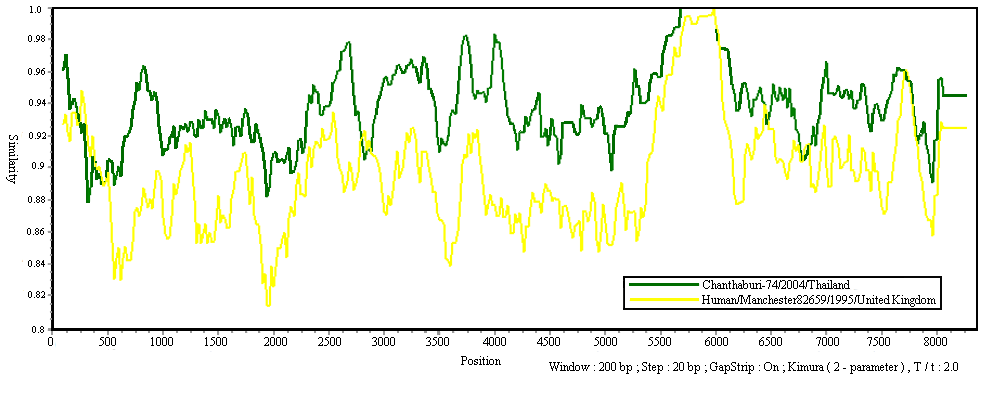

Supplement: Supplementary file 2 — Supplementary material 2 (TIFF 2352 kb) [file 11262_2016_1343_MOESM2_ESM.tif]
