# Supplementary material for: Complete genome sequence of a sapovirus from a child in Zhejiang, China
Source: Virus Genes. 2016 Apr 28;52(5):706–10. doi: 10.1007/s11262-016-1343-9 (PMC5002278; doi:10.1007/s11262-016-1343-9)
Supplement: Supplementary file 3 — Supplementary material 3 (DOC 82 kb) [file 11262_2016_1343_MOESM3_ESM.doc]

Supplementary material

Table S1 Location for each misc_feature and typical amino acid motifs in predicted viral nonstructural proteins (NS1 to NS7) and structural proteins VP1 in open reading frame 1.

| Type | Name | Start | End |
| --- | --- | --- | --- |
| misc_feature | NS1 | 1 | 68 |
| misc_feature | NS2 | 69 | 324 |
| misc_feature | NS3 | 325 | 665 |
| misc_feature | NS4 | 666 | 939 |
| misc_feature | NS5 | 940 | 1053 |
| misc_feature | NS6-NS7 | 1054 | 1721 |
| misc_feature | VP1 | 1722 | 2280 |
| Motif | GAPGIGKT | 480 | 487 |
| Motif | KGKTK | 941 | 945 |
| Motif | DDEYDE | 962 | 967 |
| Motif | GDCG | 1167 | 1170 |
| Motif | WKGL | 1211 | 1214 |
| Motif | KDEL | 1373 | 1376 |
| Motif | DYSKWDST | 1448 | 1455 |
| Motif | GLPSG | 1503 | 1507 |
| Motif | YGDD | 1551 | 1554 |
| Motif | PPG | 1740 | 1742 |
| Motif | PPG | 1854 | 1856 |
| Motif | PPG | 1944 | 1946 |
| Motif | GWS | 1999 | 2001 |

Table S2 Genbank accession number of reference nucleotide sequences used in phylogenetic analysis.

| Isolate Name | Genbank Number |
| --- | --- |
| Chanthaburi-74/2004/Thailand | AY646854.2 |
| NongKhai-50/2004/Thailand | AY646853.2 |
| NongKhai-24/2004/Thailand | AY646856.2 |
| Human/Dresden291175/Pjg-Sap01/2004/Germany | NC_006269.1 |
| chimp/IJC04/2011/Tchimpounga | KJ858686.1 |
| chimp/IJC09/2011/Tchimpounga | KJ858687.1 |
| Human/GI/Sapporo/MT-2010/1982/Japan | HM002617.1 |
| Human/GI.1/1982/Japan | U65427 |
| Hu/GI.1/Seoul/ROK62/2013/KOR | KP298674.1 |
| Human/Manchester82659/1995/United Kingdom | X86560.1 |
| Mc114 | AY237422 |
| N21/2003/Japan | AY237423 |
| Hu/Ehime643/March 2000/JP | DQ366345 |
| Human/GI.3/Stockholm318/1997/Sweden | AF194182 |
| Human/BD/697/2005/Bangladesh | GQ261222.1 |
| Human/GI.2/Parkville/1994/USA | U73124 |
| Hu/G1/BE-HPI01/DE/2012 | JX993277.1 |
| Hu/GI.2/Kecskemet/HUN3739/2008/HUN | FJ844411.5 |
| Hu/GI.2/BR-DF01/BRA/2009 | AB614356.1 |
| Zalophus_californianus/9775/2010/USA | JN420370.2 |
| Hu/Nagoya/NGY-1/2012/JPN | NC_027026.1 |
| Human/GV.1/Argentina39/Argentina | AF405715.1 |
| Hu/Ehime475/2004/JP | DQ366344.1 |
| Human/GIV.1/Hou7-1181/1990/USA | AF435811.1 |
| Hu/Angelholm/SW278/2004/SE | DQ125333 |
| Hu/Angelholm/SW314/2004/SE | DQ125334 |
| Hu/Ehime1596/1999/JP | DQ366346 |
| Hu/SV/Chiba/000671/1999/JP | AJ786349.2 |
| Ehime1107/2002/JP | DQ058829 |
| GII/PHL-TGO12-028 | KP067444.1 |
| SaKaeo-15/2004/Thailand | AY646855.2 |
| C12/2004/Japan | NC_006554.1 |
| Human/Bristol/1998/United Kindom | AJ249939.1 |
| Human/GII.1/London/1992/United Kindom | U95645 |
| Human/GII.3/cruse_ship/2000/USA | AY289804 |
| Human/GII.2/Mex340/1990/Mexico | AF435809.1 |
| 8142793/2012/USA | GZ210369.1 |
| 8124104/2012/USA | GZ179800.1 |
| 7527801/2009/USA | GP426694.1 |
| Mc10/2007/Thailand | CS790749.1 |
| Mc10/2009/Japan | HV042517.1 |
| Mc10/2003/Thailand | NC_010624.1 |
| Dog/AN210D/USA/2009 | JN387134.2 |
| Pig/GVI/OH-JJ674/2000/USA | KJ508818.1 |
| Pig/OH-JJ681/2000/USA | AY974192.2 |
| Pig/K7/2005/Japan | AB221130.1 |
| Sus_scrifa/ah-1/2009/China | JX678943.1 |
| pig/sav1/2008/CHN | FJ387164.1 |
| Pig/Gansu/CH430/2012/CHN | KF204570.1 |
| Pig/LL14/2004/USA | AY425671.1 |
| Pig/Cowden/1979/USA | KT922087.1 |
| Pig/GIII.1/PEC-Cowden/1980/USA | AF182760 |

Figure S1 A schematic diagram of sapovirus genomic organization, including putative subgenomic transcript, open reading frames (ORF1, ORF2 and ORF3), predicted viral nonstructural proteins and structural proteins VP1 and VP2. Cleavage sites shown are based on previous report [2].

Figure S2 Result of Simplot analysis. Similarity plot analysis was conducted with the strain Human/Zhejiang1/2015/China as the query sequence. Both strain Chanthaburi-74/2004/Thailand (AY646854.2) in green and strain Human/Manchester82659/1995/United Kingdom (X86560.1) in yellow were not possible recombination parents related to the query sequence.
